# Second-dose measles vaccination and associated factors among under-five children in urban areas of North Shoa Zone, Central Ethiopia, 2022

**DOI:** 10.3389/fpubh.2022.1029740

**Published:** 2022-12-09

**Authors:** Addisu Waleligne Tadesse, Degemu Sahlu, Mengistu Benayew

**Affiliations:** ^1^Department of Public Health, College of Health Science, Salale University, Fitche, Ethiopia; ^2^Department of Nursing, College of Health Science, Salale University, Fitche, Ethiopia

**Keywords:** Central Ethiopia, MCV2 vaccination, North Shoa Zone, second dose measles vaccination, under-five children

## Abstract

**Introduction:**

Measles remain a leading cause of vaccine-preventable infant mortality. In Africa, about 13 million cases and 6,50,000 deaths occur annually, with Sub-Saharan Africa having the highest morbidity and mortality. Ethiopia launched second-dose measles vaccination into the routine immunization program in the second year of life in 2019. However, little has been known about the coverage of the second-dose measles vaccine. Therefore, the purpose of this study was to assess the level of second-dose measles vaccine uptake and associated factors in North Shoa Zone, Central Ethiopia.

**Objective:**

To assess second-dose measles vaccination and associated factors among under-five children and to identify reasons for not being vaccinated in urban areas of North Shoa Zone, Central Ethiopia, 2022.

**Method:**

A community-based cross-sectional study was conducted from 1 February to 15 March 2022. The sample size was 410, and it was allocated proportionally to each *kebelle*. The study units were selected consecutively. The data were collected using structured interviewer-administered questionnaires. Four nurses were used as data collectors. Data were coded manually and entered into Epi-data Version 4.4.2.1. Frequency and cross-tabs were used for data cleaning. Data were analyzed using SPSS Version 21 software. Multicollinearity and model goodness-of-fit tests were checked. A multivariable logistic regression model at 95% CI was used to identify factors associated with the dependent variable.

**Result:**

The response rate was 90.7%. The level of second-dose measles vaccination among children in urban areas of North Shoa Zone was 42.5% [95% CI (36.8, 47.3)]. Maternal age of ≤ 25 years [AOR = 9.12: 95% CI (1.97, 42.19)], 26–30 years [AOR = 9.49: 95% CI (2.33, 38.63)], 31–35 years [AOR = 7.87: 95% CI (1.78, 34.79)]; average time mothers had been waiting for vaccination at the health facility [AOR = 3.68: 95% CI (1.33, 10.23)]; awareness about vaccine-preventable diseases [AOR = 4.15: 95% CI (1.53, 11.26)]; and awareness on recommended measles doses [AOR = 17.81: 95% CI (3.91, 81.22)] were identified as factors associated with MCV2 vaccination. The major reason (48.1%) reported by mothers for not vaccinating second-dose measles vaccine was being unaware of the need to return for second-dose measles vaccination.

**Conclusion and recommendation:**

The level of second-dose measles vaccination (MCV2) among children in urban areas of the North Shoa Zone was low. Maternal age, average time mothers had been waiting for vaccination at the health facility, awareness about vaccine-preventable diseases, recommended age for the last vaccination, and recommended measles doses were identified as factors associated with MCV2 uptake. The major reason for not vaccinating MCV2 was a lack of information (unaware of the need to return for MCV2, unaware of the need to return for MCV2, and the place and/or time of immunization unknown). Hence, enhancing awareness about vaccine-preventable diseases, shortening the average time for vaccination at the health facility by half an hour, creating an alerting mechanism for MCV2 appointments, and future studies on the effect of healthcare provider-related factors on MCV2 uptake are recommended.

## Introduction

Measles is a highly contagious vaccine-preventable respiratory disease killing an estimated 2.6 million people globally every year (1–3). It is caused by the paramyxovirus virus that spreads through respiratory droplets when an infected person coughs or sneezes (1).

Before starting the measles vaccination program, nearly 90% of children aged under 15 years were infected with measles (4). In the era of the expanded immunization program (EPI), global measles deaths declined by three-fourths from 2000 to 2014, but measles is still considered a public health emergency that requires immediate notification and rapid public health response (5).

Despite the comprehensive WHO and UNICEF measles-reduction strategy and the partnership of international organizations for measles mortality reduction, certain countries continue to face recurrent epidemics (6). Measles incidence and transmission in a population are effectively prevented by the vaccination of at least 95% of individuals with two doses of measles vaccine to ensure herd immunity, that is, the protection of everyone including those who cannot be immunized (7). Hence, over 93–95% of population immunity is required to prevent measles epidemics. The WHO recommends all children receive two doses of the measles vaccine (8, 9).

In 2009, WHO recommended countries introduce the second dose of measles vaccine (MCV2) in the routine immunization (RI) schedule once they have achieved ≥80% coverage of the first dose of measles vaccine (MCV1) at the national level for 3 consecutive years as determined by the most accurate data available (9). However, the MCV2 introduction policy was revised in April 2017, recommending that countries include MCV2 in their national vaccination schedules regardless of the level of MCV1 coverage (10). Ethiopia launched MCV2 vaccination into the routine immunization program in the second year of life. The official launching was celebrated at Wolenchity Health Center, Bosete Woreda of Oromia region on 11 February 2019.

In Africa, about 13 million cases and 6,50,000 deaths of measles occur annually, with Sub-Saharan Africa having the highest morbidity and mortality (11). Sequela of measles includes giant cell pneumonia, inclusion body encephalitis, and sub-acute sclerosing pan-encephalitis (12).

Although measles outbreak response is necessary to reduce morbidity and mortality, such activities have substantial financial and opportunity costs. In the United Kingdom, the 2012/13 measles outbreak included 2,458 reported cases; the estimated cost of the outbreak was £4.4m comprising 15% (£0.7m) of National Health Service patient treatment costs, 40% (£1.8m) public health costs, and 44% (£2.0m) societal productivity losses (13).

A study conducted in 2020 about the economic burden of measles infection in Bangladesh showed that a hospitalized and ambulatory case of measles cost $159 and $18, respectively. On average, the government spent $22 per hospitalized case of measles. At the same time, caregivers incurred $131 and $182 in economic costs, including $48 and $83 in out-of-pocket expenses in public and private not-for-profit facilities, respectively. Seventy-eight percent of the poorest caregivers faced catastrophic health expenditures compared to 21% of the richest (14).

It had been documented that measles outbreaks in Ethiopia (Keffa Zone) incur economic costs amounting to US$72.29 per case for the health sector (including outbreak response immunization campaign), and US$29.18 per case for households which equals 6% of the household median annual income. The outbreak economic cost was equal to 44% of the same year's public health expenditure for the entire Keffa Zone (15).

Although MCV2 had been introduced recently in Africa, the coverage remains low in most countries. Among 26 countries that introduced MCV2, only eight of them had coverage of more than 80% in 2015. The coverage was 60–80% in seven countries, whereas it was < 60% in eight countries (16). The study conducted in Kenya showed that MCV2 coverage was only 17.9% (17).

The mother's or caretaker's awareness of the MCV2-containing vaccine, < 30 min taken to an immunizing health facility, uptake of Pentavalent 3, and at least two doses of Vitamin A were significantly associated with the uptake of the MCV2-containing vaccine (17). However, since the introduction of MCV2 vaccination in the routine EPI program, little has been known about the coverage of MCV2, both in urban and rural areas, in Ethiopia. Zonal reports of MCV2 coverage from urban areas are low in North Shewa Zone; hence, this study was conducted to assess the coverage of MCV2 and to identify the reasons for not taking the vaccine in the urban area of North Shoa Zone, Central Ethiopia. This study could provide baseline information for programmers, planners, and stakeholders about the level of MCV2 in the North Shewa Zone. It could also provide benefits for the health office/facilities by identifying obstacles to vaccination and taking appropriate measures. This research could be used as a reference for further investigation of MCV2 in Ethiopia in the future.

## Methods and materials

### Study area, study design, and study period

A community-based cross-sectional study was conducted in selected urban areas of North Shoa Zone, Oromia, from 1 February to 15 March 2022. The zone is structured into 13 woredas and one town administration. North Shewa is bordered on the south by Addis Ababa, on the southwest by West Shewa, on the north by the Amhara Region, and on the southeast by East Shewa. Based on the 2007 Census conducted by the Central Statistical Agency of Ethiopia (CSA), this Zone has a total population of 14,31,305, of whom 7,17,552 are men and 7,13,753 women; with an area of 10,322.48 km^**2**^. There are 64 health posts, 268 health centers, and five hospitals (two general and three primaries) providing health services for 1.6 million people. The zone has been giving MCV2 since 2019 [*Source: North Shoa Health Office and https://en.wikipedia.org/wiki/North_Shoa_Zone(Oromia)*].

### Study population

All children of < 60 months with their mothers/caregivers in urban areas of North Shewa Zone were the source population. Whereas, children with ages ≥15 and < 36 months with their mothers/caregivers living in selected urban areas of north Shewa were the study population.

### Inclusion and exclusion criteria

Children aged ≥15 and < 36 months with their mothers/caregivers and who had vaccination cards with written records of vaccination dates were included in the study. Children whose appointment for second-dose measles vaccination was within the study period; absent for the third-time visit during data collection; ill mothers/caretakers who were unable to respond or were residents of < 6 months were excluded from the study.

### Sample size and sampling procedures

The sample size was determined as follows based on a single population proportion formula with a 95% level of confidence, a 5% marginal error, and *P* = 41% (WHO and UNICEF estimates of immunization coverage: 2019 revision) (18).


$$\begin{array}{l} n=\frac{z\alpha^{2}p\left(1-p\right)}{d^{2}}=\frac{1.96^{2}*0.41\left(1-0.41\right)}{0.05^{2}}=372; \end{array}$$

where *n* is the sample size; $Z_{\frac{\alpha }{2}}$ is the standardized value for the level of confidence; *p* is the proportion of MCV2 vaccination; and d is the margin of error. Considering a 10% non-response rate, the final sample size by using a single population proportion formula was 410.

The sample size for factors was determined by using EPI INFO Version 7 Stat calc by considering received penta3, time taken to nearest health facility, and received ≥ 2 doses of vitamin A (Supplementary Table 1). Hence, the sample size obtained by the proportion was higher than the sample size obtained by factors; the sample size for this study will be 410.

The total sample size was allocated to selected towns proportionally based on last year's MCV2 plan in each town. Then, from towns that had three or more *kebelles* (the lowest structural administration), we selected two *kebelles*, and one *kebelle* was selected for those that had < 3 *kebelles* randomly. Since the study population is between the ages of 15–36 months, all households had no children in this age group. Therefore, households in which participants fulfilled the inclusion criteria were selected consecutively. Public facilities such as health centers, churches, health posts, or any governmental offices had been used as the reference point to select the first household (Supplementary Figure 1).

### Variable of the study

The dependent variable was MCV2 vaccination. Maternal educational status, maternal age, knowledge, income, ANC follow-up, place of delivery, MCV1 vaccination, age of child vaccination, penta3 vaccination, vitamin A uptake, and distance to health facility were independent variables.

### Operational definition and definition of terms

**Coverage** The proportion of children aged 15–36 months who received a particular vaccine antigen (19).
**Uptake** It is the utilization of vaccination services for a particular vaccine antigen (19).
**Vaccine** It is a biological preparation that improves immunity to a particular disease (19).
**Good knowledge** Respondents who have a knowledge score of the mean and above.
**Poor knowledge** Respondents who have a knowledge score below the mean.

### Data collection procedures (instruments and personnel)

The data were collected using structured interviewer-administered questionnaires. The questionnaire was prepared by reviewing different published literature and adapted to the objective of this study (17, 19–22). The questionnaire was initially prepared in English. The English version was translated to the Amharic and Affan Oromo local languages and was translated back to English to ensure internal consistency. Six nurses were recruited to collect the data. All data collectors and supervisors had been trained for 1 day.

### Data quality control

Data quality was ensured during collection, coding, entry, and analysis. Before data collection, training and orientation were given to data collectors and supervisors. Follow-up was also made on them during the data collection period. Moreover, the questionnaire was pre-tested on 5% of samples 15 days before the actual data collection time to ensure clarity, wording, and logical sequence of the questions. In addition, the supervisors and principal investigators supervised the whole activity of the data collection process and checked the filled questionnaires every day for completeness and correctness, and necessary corrections were made timely.

### Data processing and analysis

Data were exported from EpiData Version 4.6.0.2 to SPSS version 21 statistical packages for analysis. Missing values were checked and labeled accordingly. The recording of variables was done. Descriptive statistics was used according to the type of data. Multicollinearity between the independent variables was checked. Model goodness-of-fit was checked by the Hosmer–Lemeshow statistic. Bivariate logistic regression was used to determine the association between the dependent and each independent variable by using the enter method. Those variables with a *P*-value of 0.25 and below were candidates for multivariate logistic regressions. Using backward stepwise logistic regression the predictors for second-dose measles vaccine uptake among under-five children in Fitche Town were identified. The odds ratio (crude and adjusted odds ratio) with their 95% CI was calculated. *P*-value < 0.05 was considered to decide the statistical significance.

### Ethical consideration

The study was approved by the Institutional Research Ethics Review Board Office at Salale University College of Medicine and Health Science. A formal letter was written to North Shoa Zonal Health Office and then a supporting letter was written to Fitche Town Health Office. Informed written consent was taken from each study participant after briefing the aim of the study.

## Result

### Socio-demographic characteristics of study participants

A total of 372 participants were involved in the study, which gives a response rate of 90.1%. The reason for non-participation was a refusal to be involved in the study. The average age of mothers was 29.14 years (±4.88 years). Nearly half of the mothers (48.7%) were farmers or housewives by their occupation (Table 1).

**Table 1:** Socio-demographic characteristics of mothers involved in the study of MCV2 vaccination and associated factors in urban areas of North Shoa Zone, Oromia Region, 2022.

**Characteristics**		**Frequency**	**Percentage**
Age of the mother (in years)	≤ 25	90	24.2
	26–30	153	41.1
	31–35	84	22.6
	≥36	45	12.1
Maternal educational	Unable to read and write	95	25.5
status	Primary	85	22.8
	Secondary	122	32.8
	College and above	70	18.8
Maternal occupation	Farmer/housewife	181	48.7
	Private business	68	18.3
	Governmental profession	102	27.4
	Causal laborer	21	5.6
Family monthly income	< 5,000	229	61.6
in ETB	≥5,000	143	38.4
Family size	1–3	110	29.6
	4–5	195	52.4
	≥6	67	18
Number of children alive	One	102	27.4
	Two	133	35.8
	Three and above	137	36.8

### Characteristics of children involved in the study (*n* = 372)

The majority (89%) of children had been living with both parents. About three-fourths (72.8%) of children involved in the study were between the ages of 24 and 36 months. Twenty-nine percent of children were the first child of their parents, while 36% were second order. The remaining 20.4 and 14.5% of children were in third and fourth or later order, respectively.

### Description of access and service-related factors

Only 22.8% of study participants could walk more than 20 min to arrive at the nearest health facility for the service of vaccination. The average waiting time to get service after arriving at the health facility was more than 30 min for 60.5% of study participants (Table 2).

**Table 2:** Distribution of accesses and service-related factors among study participants for MCV2 vaccination and associated factors in urban areas of North Shoa Zone, Oromia Region, 2022.

**Characteristics**		**Frequency**	**Percent**
Time to arrive at the nearest vaccination center on foot	≤ 30 min	353	94.9
	>30 min	19	5.1
Place of delivery	Home	21	5.6
	Health facility	351	94.4
History of ANC follow-up during pregnancy	Yes	352	94.6
	No	20	5.4
Waiting time to get service after arriving at health facility	≤ 30 min	225	60.5
	>30 min	147	39.5
Ever have returned back without being vaccinated	Yes	50	13.4
	No	322	86.6

### Mother's awareness and perception-related factors

In this survey, only 55.4% of mothers knew about vaccine-preventable diseases. More than three-fourths (77.2%) responded that they will take their children to the health facility for vaccination, although their child is sick (Table 3).

**Table 3:** Distribution of mothers' awareness and perception-related factors among study participants for MCV2 vaccination and associated factors in urban areas of North Shoa Zone, Oromia Region, 2022.

**Characteristics**		**Frequency**	**Percent**
Awareness about VPD of children	Yes	206	55.4
	No	166	44.6
Recommended time for first vaccination of child	From birth−4 weeks	229	61.6
	After 4 weeks	121	32.5
	Don't know	22	5.9
Recommended time for last vaccination of child	At 9 month	166	44.6
	From 15 to 24 months	173	46.5
	Don't know	33	8.9
Number of doses a child should take	One	139	37.4
	Two	170	45.7
	Don't know	33	16.9
Worrying that vaccine can a cause child to be sick	Yes	138	37.1
	No	234	62.9
Taking child for vaccination even if he/she is sick	Yes	287	77.2
	No	85	22.8

### The level of MCV2 among children in urban areas of the North Shoa Zone

**Figure 1:**
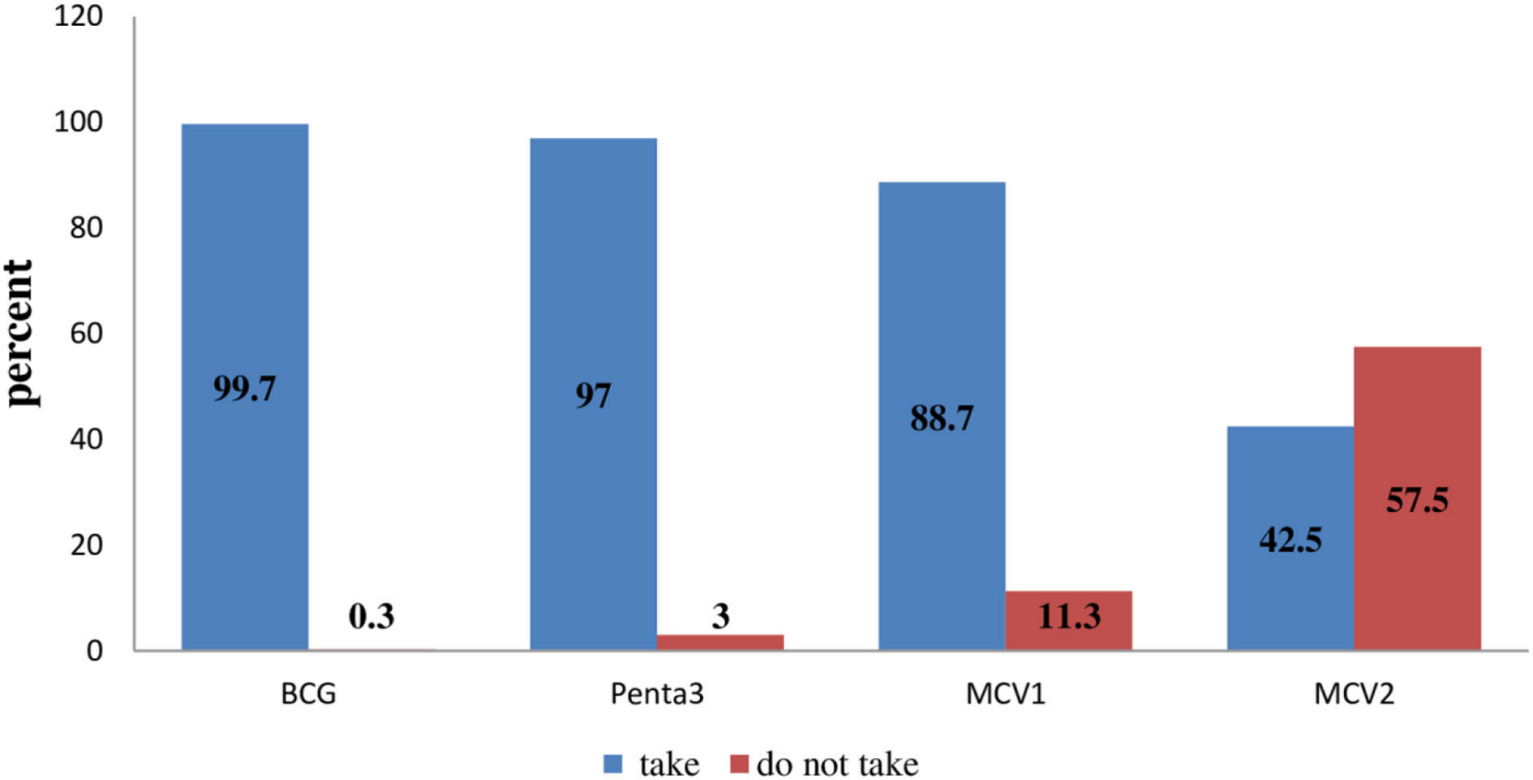
The level of MCV2 among children in urban areas of North Shoa Zone, Oromia Region, 2022.

The level of second-dose measles vaccination (MCV2) among children in urban areas of the North Shoa Zone was 42.5% [95% CI (36.8, 47.3)]. Whereas, the proportion of first-dose measles vaccination was 88.7% (Figure 1).

### Factors associated with MCV2 vaccination

In bivariate analysis, the age of the mother, maternal education, family monthly income, maternal occupation, age of the child, number of children, order of the child, with whom the child lives, the average time you have been waiting for vaccination at the health facility, returning back home without vaccination, awareness about vaccine-preventable diseases, recommended age for first vaccination, recommended age for the last vaccination, recommended measles doses, taking the child for vaccination if he/she is ill, worried vaccines can cause one's child sick, and has been vaccinated were identified as candidates for multivariable logistic regression.

In multivariable analysis, maternal age, average time mothers had been waiting for vaccination at the health facility, awareness about vaccine-preventable diseases, recommended age for the last vaccination, and recommended measles doses were identified as factors associated with MCV2 uptake. The odds of being vaccinated for MCV2 were higher among children with mothers younger than 25 years [AOR = 9.12: 95% CI (1.97, 42.19)], 26–30 years [AOR = 9.49: 95% CI (2.33, 38.63)], and 31–35 years [AOR = 7.87: 95% CI (1.78, 34.79)] compared to children with mothers older than 36 years. Children whose mothers had an average waiting time of ≤ 30 min for vaccination at the health facility were 3.7 times [AOR = 3.68: 95% CI (1.33, 10.23)] more likely to be vaccinated for MCV2 compared to waiting time of >30 min (Table 4).

**Table 4:** Bivariable and multivariable logistic regression among study participants on MCV2 vaccination and associated factors in urban areas of North Shoa Zone, Oromia Region, 2022.

**Variable**	**MCV2-status**		**COR (95% CI)**	***P*-value**	**AOR (95% CI)**
	**Yes**	**No**			
**Age of the mother**					
≤ 25	40	50	2.8 (1.24, 6.34)	0.01	9.12 (1.97, 42.19)^*^
26–30	76	77	3.46 (1.60, 7.47)	0.00	9.49 (2.33, 38.63)^*^
31–35	10	35	2.15 (0.94, 4.94)	0.07	7.87 (1.78, 34.79)^*^
≥36	10	35	1.00		
**Maternal marital-status**					
Married	149	190	2.09 (0.94, 4.63)	0.07	
Others^**+**^	9	24	1.00		
**Maternal education-level**					
Unable to read write	22	73	1.00		1.00
Primary	32	53	2.00 (1.05, 3.83)	0.04	0.46 (0.14, 1.54)
High school	61	61	3.32 (1.83, 6.01)	0.00	1.00 (0.31, 3.24)
College and above	43	27	5.28 (2.68, 10.4)	0.00	3.34 (0.90, 12.32)
**Family monthly income in ETB**					
< 5,000	84	145	1.00	0.00	
≥5,000	74	69	1.85 (1.21, 2.83)	0.00	
**Maternal occupation**					
Farmers/housewife	65	116	1.00		
Governmental workers	68	55	2.21 (1.38, 3.52)	0.00	
Business/causal laborer	25	43	1.04 (0.58, 1.85)	0.90	
**Age of child**					
24–36 months	108	163	0.68 (0.43, 1.07)	0.1	
37–48 months	50	51	1.00		
**Number of alive children**					
One	49	53	1.09 (0.65, 1.82)	0.75	
Two	46	87	0.62 (0.1.01)	0.06	
Three and above	63	74	1.00		
**Order of the child**					
First order	54	54	1.32 (0.79, 2.21)	0.29	
Second order	48	86	0.74 (0.45, 1.21)	0.23	
Third order	56	74	1.00		
**With whom the child live**					
With both parents		146	185	1.91 (0.94, 3.87)	0.07
Mother's only		12	29	1.00	
**Average time you have been waiting for vaccination at the health facility**					
≤ 30 min	85	140	0.62 (0.40, 0.94)	0.02	3.68 (1.33, 10.23)^*^
>30 min	73	74			1.00
**Returning back without vaccination**					
Yes	14	36	1.00		
No	144	178	2.08 (1.08, 4.01)	0.03	
**Age recommendation for last vaccination**					
15–24	147	26	96.63 (46.23, 202)	0.00	23.11 (4.65, 114.92)^*^
At 9th month/don't know	11	188	1.00		
**Awareness about vaccine preventable diseases**					
Yes	89	117	1.07 (0.71, 1.62)	0.75	4.15 (1.53, 11.26)^*^
No	69	97	1.00		1.00
**Recommended doses of measles**					
Two	146	24	96.3 (46.62, 199)	0.00	17.81 (3.91, 81.22)^*^
One/don't know	12	190	1.00		1.00
**Worried vaccines can cause one's child sick**					
Yes	45	93	1.00		
No	113	121	1.93 (1.25, 3.00)	0.00	
**Taking child for vaccination if he he/she is ill**					
Yes	127	160	1.38 (0.84, 2.28)	0.2	
No	31	54	1.00		
**Has been vaccinated Pneta-3**					
Yes	157	204	7.70 (0.98, 60.76)	0.05	
No	1	10	1.00		

* Significantly associated.^+^single, divorced, widowed, separated.

### Reasons for not being vaccinated

**Figure 2:**
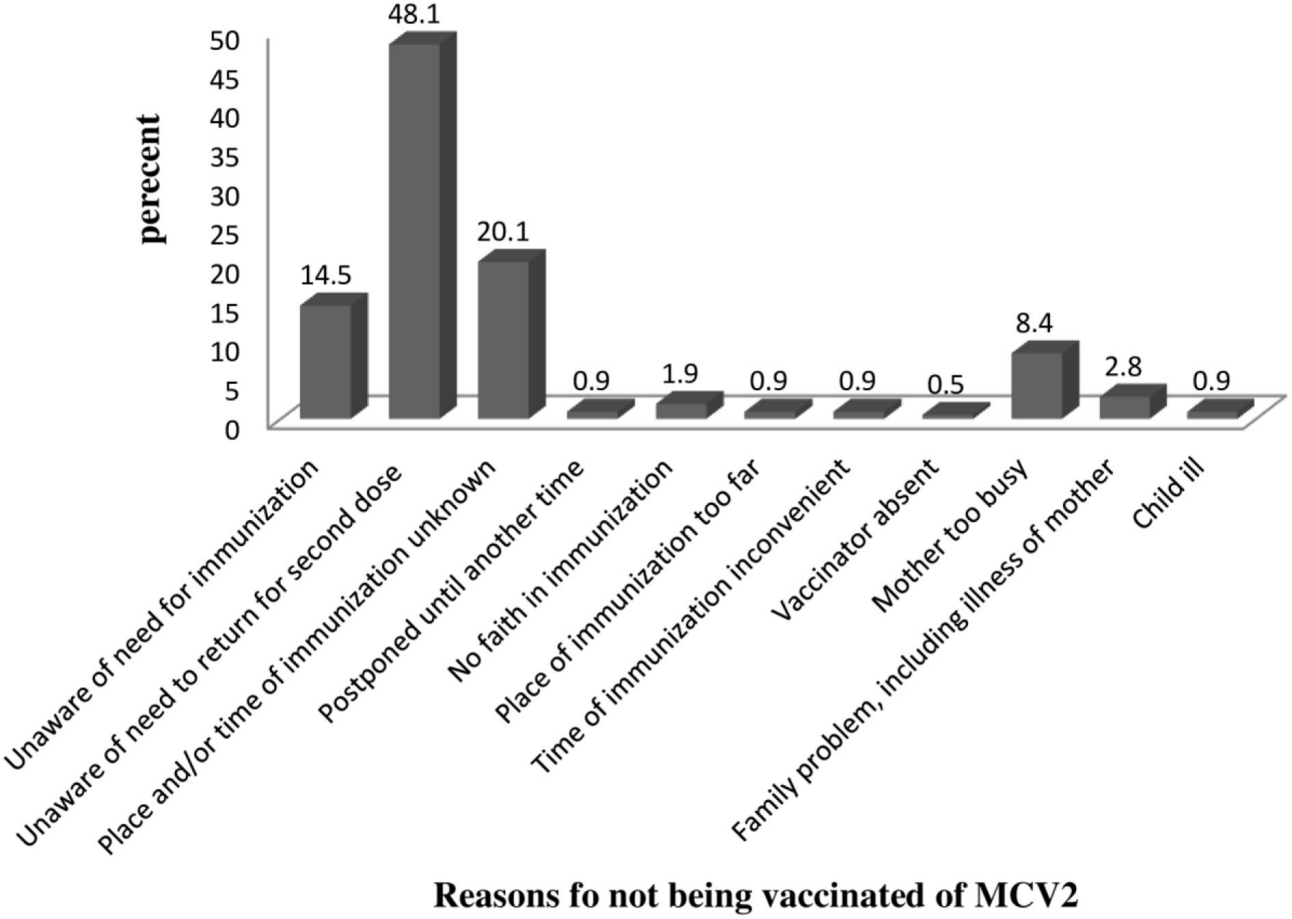
Reason for not vaccinating among study participants on MCV2 vaccination and associated factors in urban areas of North Shoa, Oromia Region, 2022.

Among 372 study participants, 214 of them had not taken MCV2. The major reason (48.1%) for not vaccinating MCV2 was being unaware of the need to return for second-dose measles vaccination. Twenty percent of mothers who did not vaccinate their child responded that they did not know MCV2 vaccination date/site (Figure 2).

## Discussion

### The level of MCV2 vaccination

The level of second-dose measles vaccination (MCV2) among children in urban areas of the North Shoa Zone was 42.5% [95% CI (36.8, 47.3)]. This finding was consistent with findings from the Mtwara district council (44.2%), Tanzania (21). However, it was much lower compared to MCV2 coverage in Tanzania in 2021 (20). Whereas, it was higher as compared to findings from Eastleigh, North Ward Nairobi County (35.5%) (19), and Kakamega County (17.9%) (17) in Kenya. The difference might be due to the recent study in Tanzania had been conducted after long years of the initiation of MCV2 which could provide the opportunity for the community to be more aware of MCV2. In addition, the recommended time for MCV2 uptake in Tanzania was with 18 months but within 15–24 months in Ethiopia. Although this long duration of time could increase coverage of MCV2, it might also increase the probability of failing to remember the appointment date as the duration between MCV1 and MCV2 ranges from 6 to 15 months in Ethiopia. The finding from this study could strengthen the justification as more than two-thirds (68.2%) of mothers responded that being unaware of the return for MCV2 or unknown time of immunization as reasons for missing MCV2.

### Access and service related factors

The time taken to access the service at the nearest health facility on foot was ≤ 30 min by the majority [(*P* = 94.9%: 95% CI (92.6, 97.3)] of study participants. This was higher compared to finding from Kenya (81.2%) (19). This might be due to variations in population distribution between the two study areas. Even though it is not clearly stated in the method parts, a study from Kenya might involve rural/semi-urban areas; whereas this study involved only urban residents. The sample size used by the Kenyan researcher was only 186, but this study involves 372 participants, therefore, this might lead to little variation.

The majority [*P* = 94.4%: 95% CI (92.2, 96.8)] of children had been delivered to the health facility. This was consistent with findings from Kenya (95.2%) (19) and Tanzania (95.2%−97.2%) (20, 21). On the other hand, 39.5% (95% CI 34.4, 45.2) of study participants responded that they will wait more than 30 min at the health facility to get the service. An indestructible number of (13.4%) mothers told that they had never returned home without getting the vaccination service. In Ethiopia, usually, BCG and measles vaccines are not open for two or three children. As a result, mothers/caregivers are obligated to have stayed in the health facility for long hours or to return home for another day; this opens an opportunity for children not to be vaccinated.

### Mothers' awareness and perception of MCV2

Only 55.4% (95% CI 50.9, 60.2) of study participants had awareness of vaccine-preventable diseases. The finding showed that this little percentage of mothers indexed at least 75% of vaccine-preventable diseases. This might be an indication that health professionals were not involved in awareness creation other than service delivery. Then again, only 46.5% (95% CI 41.7, 51.3) and 45.7% (95% CI 40.3, 50.5) of study participants were aware that the recommended age for the last vaccination is from 15 to 24 months and the number of doses of a child should take is two, respectively. This might be another indication of low awareness regarding childhood vaccination in the community.

More than one-third of [*P* = 37.1%: 95% CI (32.3, 41.9)] mothers perceived that vaccines could cause sickness for their children. This might be due to the fear of injection, as most vaccines have been given in intramuscularly or subcutaneously which creates a painful feeling. In addition, some mild side effects of vaccines might be the triggering factors for this perception. Also, 22.8% (95%: 18.5, 28.2) of mothers perceived that they would not take to vaccination if their child got the sickness. This might arise from some symptoms, like fever, usually, and the healthcare workers postponed the vaccination date till the child is improved from the illness.

### Factors associated with MCV2 non-uptake

Maternal age, average time mothers had been waiting for vaccination at the health facility, awareness about vaccine-preventable diseases, recommended age for the last vaccination, and recommended measles doses were identified as factors associated with MCV2 uptake. The odds of being vaccinated for MCV2 were higher among children with mothers younger than 25 years [AOR = 9.12: 95% CI (1.97, 42.19)], 26–30 years [AOR = 9.49: 95% CI (2.33, 38.63)], and 31–35 years [AOR = 7.87: 95% CI (1.78, 34.79)] compared to children with mothers older than 36 years.

Children whose mothers had an average waiting time of ≤ 30 min for vaccination at the health facility were four times [AOR = 3.68: 95% CI (1.33, 10.23)] more likely to vaccinate their child compared to waiting time of >1/2 h. This was supported by findings from Northwest Ethiopia and Tanzania which showed that long waiting at the health facility for vaccination services led to missing vaccination (21, 23).

Mothers who were aware of vaccine-preventable disease were four times [AOR = 4.15: 95% CI (1.53, 11.26)] more likely to vaccinate their children for MCV2. In addition, mothers who were aware of the number of measles doses recommended during the childhood period were more likely to vaccinate their children [AOR = 17.81: 95% CI (3.91, 81.22)]. This might be due to the that the more understanding of vaccine-preventable diseases, the higher intention mothers could have to vaccinate their children. Evidence also supported that having higher awareness about vaccine-preventable diseases and vaccine among mothers leads to a high probability of vaccinating their children (17).

Among 372 study participants, 214 had not taken MCV2. The major reason for not vaccinating MCV2 was the lack of information (unaware of the need to return for MCV2 = 48.1%, unaware of the need to return for MCV2 = 14.5%, and the place and/or time of immunization unknown = 20.1%). This was supported by findings from Kenya (17). This can be explained by that MCV2 is recently introduced in a routine immunization program; as a result, most mothers might not be aware of MCV2. The COVID-19 pandemic might contribute to the diversion of attention away from child healthcare.

### Strengths and limitations of the study

This was an eye-opening study in Ethiopia and included variables that had not been addressed by studies from other African countries. Not including healthcare provider-related factors was the major limitation. As the study was cross-sectional, the evidence might not be strong enough.

## Conclusion and recommendation

### Conclusion

The level of second-dose measles vaccination (MCV2) among children in urban areas of the North Shoa Zone was low. Maternal age, average time mothers had been waiting for vaccination at the health facility, awareness about vaccine-preventable diseases, recommended age for the last vaccination, and recommended measles doses were identified as factors associated with MCV2 uptake. The major reason for not vaccinating MCV2 was the lack of information (unaware of the need to return for MCV2, unaware of the need to return for MCV2, and the place and/or time of immunization unknown).

### Recommendation

For healthcare workers and health facilities

- Enhancing awareness about vaccine-preventable diseases, including recommended doses of measles and recommended appropriate age for measles vaccination, is important.
- Attention is required for children with mothers older than 36 years.
- Shortening the average time for vaccination at the health facility by half an hour should be encouraged as the best experience.

For mothers or family members

- Other family members should help mothers during vaccination day, as being busy due to work was one of the major obstacles to missing vaccination for MCV2.

For researchers and/or government officials

- The alerting mechanism for MCV2 appointments or accessible ways should be developed
- Researchers are recommended to study the effect of healthcare provider-related factors on MCV2-uptake.

## Data availability statement

The original contributions presented in the study are included in the article/Supplementary material, further inquiries can be directed to the corresponding author.

## Ethics statement

The studies involving human participants were reviewed and approved by Salale University Institutional Review Board (SlU-IRB). The patients/participants provided their written informed consent to participate in this study.

## Author contributions

AT: conceptualization, data curation, formal analysis, investigation, methodology, project administration, resources, software, supervision, validation, visualization, writing—original draft, and writing—review and editing. DS: data curation, investigation, project administration, supervision, validation, visualization, and writing—review and editing. MB: data curation, investigation, project administration, supervision, validation, visualization, and writing—review and editing. All authors contributed to the article and approved the submitted version.
