# Supplementary material for: Second-dose measles vaccination and associated factors among under-five children in urban areas of North Shoa Zone, Central Ethiopia, 2022
Source: Front Public Health. 2022 Dec 9;10:1029740. doi: 10.3389/fpubh.2022.1029740 (PMC9780268; doi:10.3389/fpubh.2022.1029740)
Supplement: Supplementary file 1 [file Data_Sheet_1.docx]

S1 Table: Calculated sample size for factors using two populations proportions by Open Epi version7

| Assumptions | Variables | | |
| --- | --- | --- | --- |
|  | received pentavalent 3, | Time taken to nearest health facility | received ≥ 2 doses of vitamin A |
|  | - Two-sided CL=95% - Power = 80% - Ratio of Unexposed to Exposed = 1 - % of outcome in unexposed group = 4.88% - % of outcome in exposed group = 20.04 - OR = 4.88 | - Two-sided CL=95% - Power = 80% - % of outcome in unexposed group = 14.19% - % of outcome in exposed group = 35.35 - OR=3.31 | - Two-sided CL=95% - Power = 80% - % of outcome in unexposed group = 9.09% - % of outcome in exposed group = 39.3963 - OR=4.52 |
| Calculated n | 174 | 148 | 74 |
| Final n (10%  contingency) | 192 | 163 | 82 |
| References | (20) | (20) | (20) |

##

## Questioners

### Information Sheet

Good morning/afternoon dear participant! My name is _________________. I am working as a data collector for the study being conducted in Fitche Town to assess second dose measles vaccination and associated factors among under-five children in urban areas of North Shewa Zone, 2021: by Addisu Walelign and his colleagues. I kindly request you to lend me your attention to explain the study and how being you selected as the study participant.

Title of the Research Project: second dose measles vaccination and associated factors among under-five children in urban areas of North Shewa Zone, 2021

Name of the Sponsor: Salale University

**Purpose of the Research Project**: To assess second dose measles vaccination and associated factors among under-five children in urban areas of North Shoa Zone, 2021

**Procedure:** For this study selected areas in urban areas of North Shewa are included. Interviewer-administered questioners are provided to participants and they will answer to questions; then, the data will be collected by the assigned data collectors.

**Risk and Benefits:** The study has no direct benefit/payments for those study participants but they may be indirectly beneficial if the result utilized by planners. North Shoa Health Office, Fitche Town Health Office will get the final result of the study. There is no risk due to participating in this study.

**Confidentiality**: No need of registering your name; therefore, the information you gave will be kept confidential. All information collected will be kept confidential and destroyed two years after the end of the project. No other persons besides the research team will see it.

**Right to Refusal or Withdraw**: To start data collection, approval of the individual participant is required. If you are not willing to participate you can refuse.

**Person to contact**: If you have any further questions or would like to receive further information about the project, please contact:

Name: Addisu Walelign: - Email: [addisuwalelign21@gmail.com](mailto:addisuwalelign21@gmail.com) (Principal Investigator)

Cell Phone: +251924462429

Degemu Sahilu:- [kergadegemu@gmail.com](mailto:kergadegemu@gmail.com) (co-investigator)

Moblie: +251915737963

Thank you for taking the time to give the Information Sheet, and asking any questions that you might have had.

**1. Family Background**

| **101** | Interviewee relationship | 1. Mother 2. Father 3. Grandmother 4. Grandfather 5. Siblings > 18 years | |
| --- | --- | --- | --- |
| **102** | Mother’s Age (in years) | **_______________________** | |
| **103** | Maternal education status | 1. Unable to read and write 2. Primary (1-8) 3. Secondary (9-12) 4. College and above | |
| **104** | Mother’s occupation | 1. Farmers/Housewife 2. Business 3. Government professional 4. Casual labourer | |
| **105** | Marital status of mother? | 1. Single 2. Married 3. Separated 4. Divorced 5. Widowed | |
| **106** | What is the mother’s average monthly income in ETB? | 1. <5,000 2. 5,000-10,000 3. 10,001-20,000 4. 20,000-50.000 5. >50,000 | |
| **107** | Family size | _____________________________ | |
| **108** | Number of parity | ______________________________ | |
| **109** | How many alive children are there? | ______________________________ | |
| **2. Characteristics of the child** | | | |
| **201** | Age of the child | __________________________ | |
| **202** | Sex of the child | 1. Male 2. Female | |
| **203** | Order of the child | 1. 1^st^ child 2. 2^nd^ child   3. 3^rd^ child 4. 4^th^ and later | |
| **204** | With whom does child live? | 1. Both parents 2. Mothers only   3. Fathers only 4. Others(_________) | |
| **3. Health Service and Access related factors** | | | |
| **301** | On foot how long do you take to reach the nearest immunization center? | _______ Minutes. | |
| **302** | Where was the child delivered? | 1. Home 2. Health facilities | |
| **303** | Place of vaccination | 1. Hospital 2. Health center 3. Health post 4. Private health facilities | |
| **304** | Did the mother attend antenatal care during pregnancy of the current child? | 1. Yes 2. No 3. Don’t Know | If your answer is “No/Don’t know” skip to question number 306 |
| **305** | If yes for question 306, how many times? | 1. One time 2. two times 3. three times 4. four times and above | |
| **306** | Waiting time for vaccination? | 1. ≤ 30 minutes 2. > 30 minutes | |
| **307** | Have you ever turned without getting vaccination? | 1. Yes 2. No | |
| **308** | Have schedules ever been canceled or postponed? | 1. Yes 2. No | |
| **4. Mother’s perception related factors question** | | | |
| **401** | Do you know vaccine-preventable diseases? | 1. Yes(_____________________________________________________________) 2. No | |
| **402** | Do you know at what age should the child start vaccination? | 1. Yes (____________) 2. No | |
| **403** | Do you know at what age should the child finish vaccination? | 1. Yes (____________________) 2. No | |
| **404** | How many doses are needed for measles vaccination within two years old? | 1. One 2. Two 3. I don’t know | |
| **405** | Do you worried vaccines can cause your child sick? | 1. Yes 2. No | |
| **406** | Do you take your child for vaccination if he/she is sick? | 1. Yes 2. No | |

**5. Request the interviewee to bring the child vaccination card for the youngest child and ask the following questions.**

| **501** | **Child Immunization** | | | |
| --- | --- | --- | --- | --- |
|  | Antigen | Status | Date given | Remark |
|  | BCG | 1. Yes 2. No | ___/____/____ |  |
|  | MCV2 | 1. Yes 2. No |  |  |
|  | MCV1 | 1. Yes 2. No |  |  |
|  | Penta3 | 1. Yes 2. No |  |  |
|  | PCV3 | 1. Yes 2. No |  |  |
|  | OPV3 | 1. Yes 2. No |  |  |
|  | Vit. A at 6 months | 1. Yes 2. No |  |  |
|  | Vit. A at 12 months | 1. Yes 2. No |  |  |
|  | Vit. A at 18 months | 1. Yes 2. No |  |  |
|  | Vit. A at 24 months | 1. Yes 2. No |  |  |
| **502** | Has your child ever suffered from measles? | 1. Yes 2. No |  |  |
| **503** | If yes for question no 502, at what age? | __________ |  | |

**6. Reasons for Measles Second Dose Immunization Failure**

**Note:** Ask only one question ‘why the child was not given second dose of measles vaccine and circle appropriately.

| **Category** | **Reason** |
| --- | --- |
| Lack of information | Unaware of need for immunization |
|  | Unaware of need to return for second dose |
|  | Place and/or time of immunization unknown |
|  | Fear of side reactions |
|  | Wrong ideas about contraindications |
| Lack of motivation | Postponed until another time |
|  | No faith in immunization |
|  | Rumors |
|  | Cultural/ religious reasons |
| Obstacles | Place of immunization too far |
|  | Time of immunization inconvenient |
|  | Vaccinator absent |
|  | Vaccine not available |
|  | Mother too busy |
|  | Family problem, including illness of Mother |
|  | Child ill |
|  | Long waiting time |
| others | _____________________________ |

1. **Family Background**

| **101** | የተሳታፊው/የተጠያቂው ዝምድና | 1. እናት 2. አባት 3. ሴት አያት 4. ወንድ አያት 5. እድሜው ከ 18 ዓመት በላይ የሆነ ቤተሰብ | **Remark** |
| --- | --- | --- | --- |
|  |  |  |  |
| **102** | የእናት እድሜ |  |  |
| **103** | የእናት የትምህርት ሁኔታ | 1. ያልትማሩ 2. የመጀመሪያ ደረጃ(1-8) 3. ሁለተኛ ደረጃ(9-12 4. ኮሌጅና ከዚያ በላይ |  |
| **104** | የእናት የስራ ሁኔታ | 1. እርሶአደር/የቤት እመቤት 2. የንግድ ስራ 3. የመንግስት ሰራተኛ 4. የቀን ሰራተኛ |  |
| **105** | የእናት የጋብቻ ሁኔታ | 1. ያገባች 2. ያላገባች 3. ተለያይተው የሚኖሩ 4. የተፋታች 5. ባላ የሞተባት |  |
| **106** | ወርሃዊ የገቢ መጠን በብር | 1. <5000 2. 5000-10000 3. 10001-20000 4. 20000-50000 5. >50000 |  |
| **107** | እናት የወለደቻቸው ልጀች ብዛት |  |  |
| **108** | ከተወለዱት ውስጥ በህይወት ያሉ የልጆች ብዛት | ____________________ |  |
| **109** | ስለልጆች ክትባት ሰምተው ያውቃሉ? | 1. አዎ 2. አልሰማሁም | **መልስዎ “አልሰማሁም” ከሆነ ወደ ጥያቄ ቁጥር 111 ይሻገሩ** |
| **110** | ለጥያቄ ቁጥር 109 መልስዎ አዎ ከሆነ መረጃውን ከየት ነው የሰሙት? | 1. ከጤና ባለሙያ 2. ከአካባቢ መሪ 3. ከጎረቤት 4. ከራደዮ/ቴሌቪዥን 5. ከሌላ (ይግለጹ_____________________) | |
| **111** | ከመኖሪያ ቤትዎ እስከ ክትባት ቦታ ምን ያክል ይወስዳል? | _______ ደቂቃ |  |
| **112** | ልጅዎ የት ነው የተወለደው? | 1. ቤት 2. ጤና ተቀም |  |
| **113** | የሕጻኑ ዕድሜ ምን ያክል ነው (በወር): | (የተወለደበት ቀን) __/___/_____ | |
| **114** | ህጻኑ ስንተኛ ልጅዎ ነው: | _____ |  |
| **115** | የልጅዎ ጾታ: | 1. ወንድ 2. ሴት |  |
| **116** | እናት የእርግዝና ክትትል አድርገው ነበር? | 1. አዎ 2. አልተከታተሉም 3. አላውቅም | **ምልስዎ “አልተከታተሉም” ወይም አላውቅም ከሆነ ወደ ጥያቄ 117 ይሻገሩ** |
| **117** | ለጥያቄ ቁጥር 116 መልስዎ አወን ከሆነ ምን የህል ጊዜ ተከታተሉ | 1. አንድ ጊዜ 2. ሁለት ጊዜ 3. ሶስት ጊዜ 4. አራት ጊዜና ከዚያ በላይ |  |
| **118** | እናት በርግዝና ወቅት የመንጋጋ ቆልፍ ክትባት ወስዳለች? | 1. አዎ 2. አልዎሰደችም 3. አላውቅም |  |

1. **Ask the interviewee to bring the mother family guide Booklet for the youngest child and ask the following questions.**

| **201** | የቤተሰብ መምሪያ ደብተር አለዎት | 1. አዎ 2. የለም | | |  | |
| --- | --- | --- | --- | --- | --- | --- |
| **202** | የህጻናት ክትባት መረጃ |  | | | | |
|  | የክትባት አይነት | | የክትባት ሁኔታ | የተከተቡበት ቀን | | Remark |
|  | BCG/ቢሲጂ | | 1. አዎ 2. አልወሰደም | ___/____/____ | |  |
|  | MCV2/ የኩፍኝ ክትባት 2 | | 1. አዎ 2. አልወሰደም |  | |  |
|  | MCV1/የኩፍኝ ክትባት 1 | | 1. አዎ 2. አልወሰደም |  | |  |
|  | Penta3/ፔንታቫለንት 3 | | 1. አዎ 2. አልወሰደም |  | |  |
|  | PCV3/የሳምባ ምች ክትባት | | 1. አዎ 2. አልወሰደም |  | |  |
|  | OPV3/የፖሊዮ(የልጅነት ልምሻ) ክትባት | | 1. አዎ 2. አልወሰደም |  | |  |
|  | Vitamin A (ቫይታሚን ኤ) at 6 months | | 1. አዎ 2. አልወሰደም |  | |  |
|  | Vitamin A (ቫይታሚን ኤ)at 12 months | | 1. አዎ 2. አልወሰደም |  | |  |
|  | Vitamin A (ቫይታሚን ኤ)at 18 months | | 1. አዎ 2. አልወሰደም |  | |  |
|  | Vitamin A (ቫይታሚን ኤ)at 24 months | | 1. አዎ 2. አልወሰደም |  | |  |
| **203** | ከልጅዎ ውስጥ የኩፍኝ ክትባት ታሞ የሚያውቅ አለ | | 1. አዎ 2. የለም |  | |  |
| **204** | ለጥያቄ ቁጥር 116 መልስዎ አወን መቼ(ዕድሜው) | | ________________ |  | | |

1. **የሚከተሉት ጥያቄዎች ልጆች ሁለተኛ የኩፈኝ ክትባት (**MCV-2) ያልዎሰዱበትን ምክንያት የሚዳስሱ ናቸው

**Note:** Ask only one question ‘why the child was not given second dose of measles vaccine and circle appropriately.

| **መደብ/Category/** | **ክትባቱን ያልዎሰዱበት ምክንያት** |
| --- | --- |
| የመረጃ እጥረት/ክፍተት | ስለክትባቱ አስፈላጊነት ግንዛቤ አለመኖር |
|  | ለሁለተኛ የኩፍኝ ክትባት ድጋኔ መምጣት እነዳለባቸው አለማወቅ |
|  | ክትባቱ የሚሰጥበትን ቦታ ወይም ጊዜ አለማወቅ |
|  | የክትባቱን የጎንዮሽ ጉዳት መፍራት |
|  | ክትባት መውሰድ ስለመከልከል(contraindications) የተሳሳተ ግንዛቤ |
| ተነሳሽነት አለመኖር | ቀጠሮውን ወደሌላ ቀን ስላለፈ |
|  | በክትባቱ እምነት ማጣት |
|  | አሉባልታዎች |
|  | ባህል/ሃይማኖት ስለማይፈቅድ |
| መሰናክሎች | የክትባት ቦታ መራቅ |
|  | ክትባቱ የሚሰጥበት ሰአት ምቹ አለመሆን |
|  | ክትባቱን የሚሰጡት ባለሙያዎች አለመኖር |
|  | ክትባት አለመኖር |
|  | እናት ሳራ ሰለበዛባት |
|  | ቤተሰባዊ ችግር (የእናት መታመም) |
|  | ልጁ ስለታመመ |
|  | ክትባት ቦታ ለረጅም ሰአት መቆየት |
| ሌሎች | _____________________________ |

1. እናት/ተንከባካቢ ስለኩፍኝ ክትባት ያላቸው ዕውቀት

| 301 | ክትባት የት እንደሚሰጥ ያዉቃሉ? | 1. አዎ (ይጥቀሱ________)  2. አላውቅም | | |
| --- | --- | --- | --- | --- |
| 302 | የኩፍኝ ክትባት የሚከላከላችዉ በሽታዎች የትኞቹ ናቸው | 1. ቲቢ/የሳምባ ነቀርሳ  2. ፖሊዮ/የልጅነት ልምሻ  3. ቴታነስ (መንጋጋ ቆልፍ, ዲፍተሪያ(diphtheria) እና ትክትክ (pertussis)  4. ኩፍኝ | | |
| 303 | የኩፍኝ በሽታ ተላላፊ ነውን? | 1. አዎ 2. አይደለም 3. አላውቅም | | |
| 304 | የኩፍኝ ክትባት በመደበኛ የክትባት መርሃ-ግብር ይካተታል? | 1. አዎ 2. አይካተትም 3. አላውቅም | | |
| 305 | የኩፍኝ ክትባት አስፈላጊ ነው ብለው ያምናሉ? | 1. አዎ 2. አይደለም 3. አላውቅም | | |
| 306 | የኩፍኝ ክትባት አገልግሎት ነጻ ነውን? | 1. አዎ 2. አይደለም 3. አላውቅም | | |
| 307 | ህጻናት የኩፍኝ ክትባት እንዳይወስዱ (እንዳይከተቡ) ሊድረግ ይችላልን ? | 1. አዎ 2. አይደለም 3. አላውቅም | | መልስዎ “አይደለም/አላውቅም” ከሆነ ወደጥያቄ 308 ይሻገሩ |
| 308 | ለጥያቄ ቁጥር 307 መልስዎ አዎ ከሆነ ከሆነ የማይሰጥበትን ሁኔታ ይጥቀሱ |  | | |
| 309 | ልጆች የኩፍኝ ክትባት መውሰድ ያለባቸው ስንት ጊዜ ነው? | 1. አንድ ጊዜ 2. ሁለት ጊዜ 3. ሶስት ጊዜ 4. አላውቅም | መልስዎ አዎ አላውቅም ከሆነ ወደ ጥያቄ ቁጥር 312 ይለፉ | |
| 310 | የመጀመሪያ ዙር ክትባት መቼ ነው መሰጠት ያለበት | 1.ስድሰተኛ ወር 2.ዘጠነኛ ወር 3. 15ኛ ወር 4. አላውቅም | | |
| 311 | የሁለተኛ ዙር ክትባት መቼ ነው መሰጠት ያለበት | 1.ስድሰተኛ ወር 2.ዘጠነኛ ወር 3. 15ኛ ወር 4. አላውቅም | | |
| 312 | ከኩፍኝ ክትባት በኀላ መጠነኛ ትኩሳት ሊከሰት ይችላል? | 1. እውነት 2. ሀሰት 3.አላውቅም | | |
| 313 | ህጻናት ክትባት ከተከተቡ በኀላ ቢያንስ ለ30 ደቂቃዎች ከባለሙያ መራቅ የለባቸውም? | 1. እውነት 2. ሀሰት 3. አላውቅም | | |
