# Supplementary figures and images for: Second-dose measles vaccination and associated factors among under-five children in urban areas of North Shoa Zone, Central Ethiopia, 2022
Source: Front Public Health. 2022 Dec 9;10:1029740. doi: 10.3389/fpubh.2022.1029740 (PMC9780268; doi:10.3389/fpubh.2022.1029740)

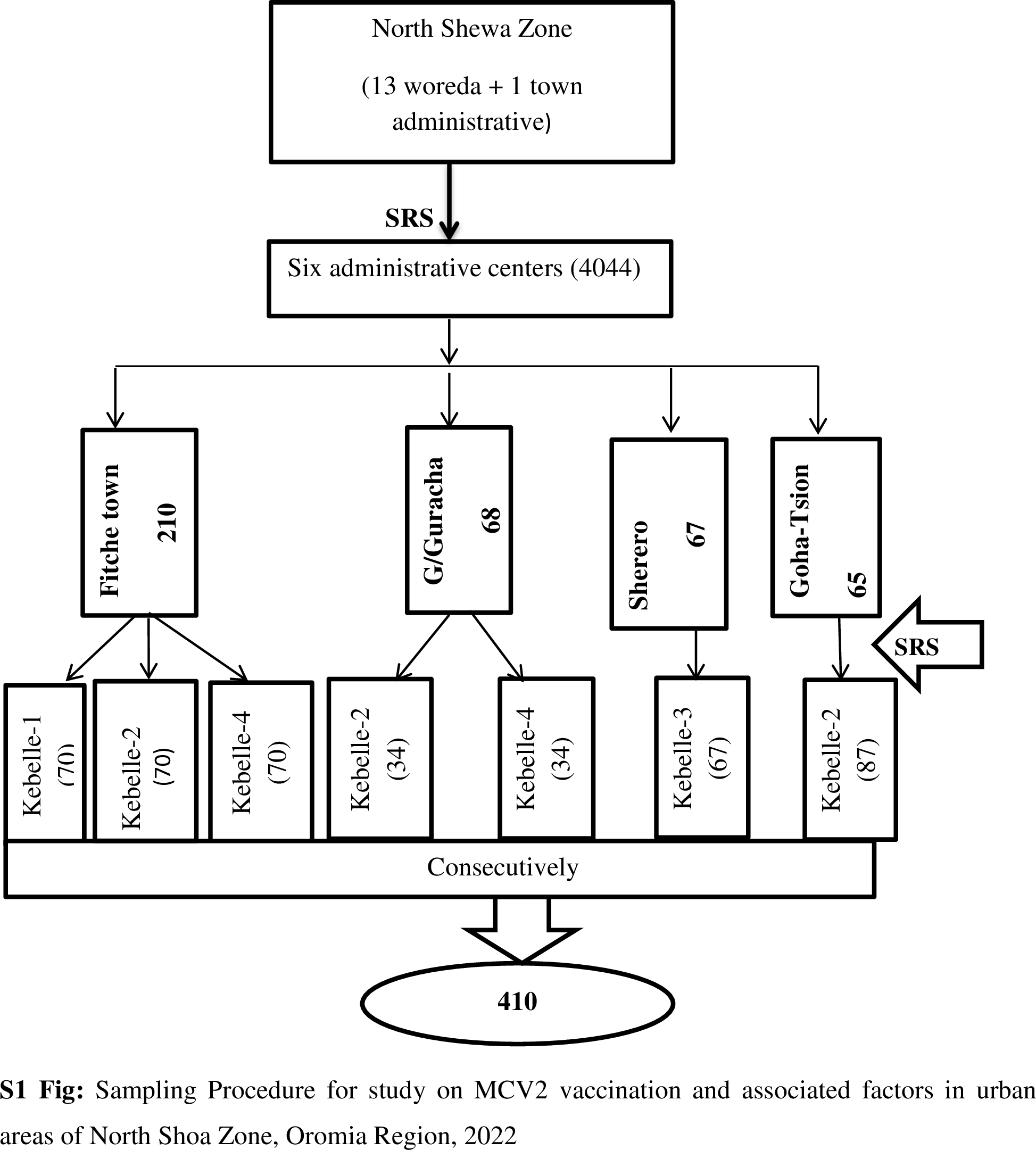

Supplement: Supplementary file 2 [file Image_1.tif]
